# The implementation of the coaching on lifestyle (CooL) intervention: lessons learnt

**DOI:** 10.1186/s12913-019-4457-7

**Published:** 2019-09-14

**Authors:** Celeste van Rinsum, Sanne Gerards, Geert Rutten, Madelon Johannesma, Ien van de Goor, Stef Kremers

**Affiliations:** 1 Department of Health Promotion, NUTRIM School of Nutrition and Translational Research in Metabolism, Maastricht University, P.O. Box 616, 6200 MD Maastricht, The Netherlands; 2 Faculty of Sciences and Engineering, University College Venlo, Maastricht University, P.O. Box 8, 5900 AA Venlo, The Netherlands; 3 Health Insurance Company CZ, P.O. 90152, 5000 LD Tilburg, The Netherlands; 4 Department Tranzo, Tilburg School of Social and Behavioral Sciences, Tilburg University, P.O. Box 90153, 5000 LE Tilburg, The Netherlands

**Keywords:** Lifestyle, Coaching, Overweight, Obesity, Combined lifestyle intervention, Implementation process

## Abstract

**Background:**

Combined lifestyle interventions (CLIs) are designed to help people who are overweight or obese maintain a healthy new lifestyle. The CooL intervention is a CLI in the Netherlands, in which lifestyle coaches counsel adults and children (and/or their parents) who are obese or at high risk of obesity to achieve a sustained healthier lifestyle. The intervention consists of coaching on lifestyle in group and individual sessions, addressing the topics of physical activity, dietary behaviours, sleep, stress management and behavioural change. The aim of this study was to evaluate the implementation process of the Coaching on Lifestyle (CooL) intervention and its facilitating and impeding factors.

**Methods:**

Mixed methods were used in this action-oriented study. Both quantitative (number of referrals, attendance lists of participants and questionnaires) and qualitative (group and individual interviews, observations, minutes and open questions) data were collected among participants, lifestyle coaches, project group members and other stakeholders. The Consolidated Framework for Implementation Research was used to analyse the data.

**Results:**

CooL was evaluated by stakeholders and participants as an accessible and useful programme, because of its design and content and the lifestyle coaches’ approach. However, stakeholders indicated that the lifestyle coaches need to become more familiar in the health care network and public sectors in the Netherlands. Lifestyle coaching is a novel profession and the added value of the lifestyle coach is not always acknowledged by all health care providers. Lifestyle coaches play a crucial role in ensuring the impact of CooL by actively networking, using clear communication materials and creating stakeholders’ support and understanding.

**Conclusion:**

The implementation process needs to be strengthened in terms of creating support for and providing clear information about lifestyle coaching. The CooL intervention was implemented in multiple regions, thanks to the efforts of many stakeholders. Lifestyle coaches should engage in networking activities and entrepreneurship to boost the implementation process. It takes considerable time for a lifestyle coach to become fully incorporated in primary care.

**Trial registration:**

NTR6208; date registered: 13–01-2017; retrospectively registered; Netherlands Trial Register.

**Electronic supplementary material:**

The online version of this article (10.1186/s12913-019-4457-7) contains supplementary material, which is available to authorized users.

## Background

An increasing proportion of the adult Dutch population is now overweight or obese (49.9 and 14.2%, respectively) [1]. The prevalence of overweight or obesity among children and adolescents has also increased (to 13.3 and 2.8%, respectively, in 2017). Combined lifestyle interventions (CLIs) aim to help people who are overweight or obese change their physical activity level and dietary behaviours and maintain the new healthier lifestyle [2, 3]. However, many interventions have failed to translate research outcomes to real-world settings, due to unsuccessful or incomplete implementation [4, 5]. Implementation of CLIs may benefit from process evaluation, as this provides insight into the implementation process. It also helps to understand the results of the intervention and the success factors influencing both the intervention and its implementation [6].

The implementation process of various types of CLI has been evaluated [7–14]. The results of many studies show too little multidisciplinary collaboration between important stakeholders, and professionals having insufficient skills and time to give participants the best possible guidance [10, 11, 15, 16]. One important barrier stopping participants from attending CLIs was that health insurers refused to cover all costs [10]. Furthermore, previous studies have shown that long-term coaching is needed to maintain lifestyle changes [2, 12, 17, 18].

The Coaching on Lifestyle (CooL) intervention was developed based on previous research findings and addresses the barriers for implementation, outlined before. In this CLI, lifestyle coaches counsel, in separate groups, children and adults who are obese or at high risk of obesity. A lifestyle coach counsels a group of participants in the longer term, on average for 6 to 8 months. Lifestyle coaching encompasses integrating and addressing all major behavioural areas linked to obesity and lifestyle, i.e. physical activity, dietary behaviours, sleep, stress management and the umbrella topic of behavioural change. The essence of lifestyle coaching does not lie in its focus on the role of the professionals, nor in giving advice or directing participants. Instead it focuses on stimulating participants to take the lead and define their personal goals, guided by means of an autonomy-supportive coaching style of the lifestyle coaches [17]. This means that the coaches first provide some basic knowledge about healthy choices, such as variation of food, conscious eating and portion sizes. Where after, participants can make their own choices and actions, for example going to work by bicycle twice a week. Furthermore, various evidence-based behaviour change techniques and approaches are incorporated in the intervention, such as goal setting, implementation intentions, ownership and peer support (see also [19]). The lifestyle coach can act as a single point of contact for the participants regarding their lifestyle goals. The coach takes on the role of linchpin in the participants’ care provider network. The intervention is reimbursed by health insurance companies and therefore free of charge for participants.

Since the trained lifestyle coach is not yet an established professional primary care, a comprehensive implementation evaluation is required, taking into account factors that may be encountered during the implementation process. The research question of the current study was: How was the CooL intervention implemented and what were facilitating and impeding factors?

**Fig. 1:**
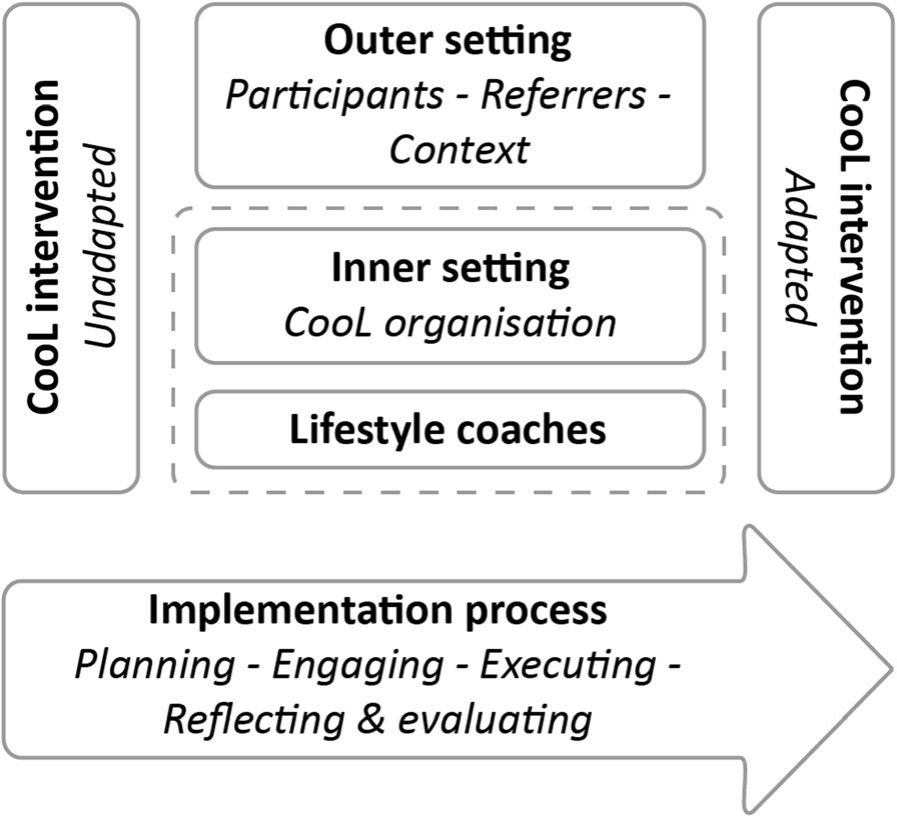
Schematic overview of the different implementation domains of the CooL intervention, based on the Consolidated Framework for Implementation Research (CFIR) [4]

The results are described using the Consolidated Framework for Implementation Research (CFIR). This framework is a synthesis of existing implementation theories and it includes constructs of effective implementation [4]. These constructs are clustered in five domains, reflecting the characteristics of implementing an intervention. The CFIR was slightly modified to make it suitable to evaluate the CooL intervention (see Fig. 1). The following key concepts of CFIR were operationalised: the unadapted and adapted intervention (CooL intervention), the process by which implementation is carried out (planning, engaging, executing, reflecting and evaluating), the inner setting (the organisation that implements the intervention: CooL organisation), the outer setting (participants, referrers and context) and the lifestyle coaches who carry out the intervention (defined in CFIR as ‘individuals’). A successful implementation process focuses on the use of the intervention by the lifestyle coaches and the inner setting. Changes in the contextual outer setting are assumed to influence both the inner setting and the implementation process. In the outer setting we also refer to stakeholders in the participants’ care provider network. It also shows that an intervention may evolve and be adapted to local preferences during the implementation process.

## Methods

### CooL intervention

The lifestyle coach leads the CooL programme, which consists of individual sessions and group sessions (see Additional file 1: Table S3). The programme targets Dutch-speaking individuals living in the Netherlands, aged 4 years and older, who are obese (BMI ≥ 30) or at high risk of obesity (i.e., were overweight (BMI ≥ 25) and at increased risk of cardiovascular diseases or type 2 diabetes mellitus) [20–22]. There are separate programmes for children, adolescents and adults. Children and adolescents are described as the same group, because of the small numbers in the programmes. Major themes are physical activity, dietary behaviours, sleep, stress management and behavioural change. The aim is to change the lifestyle pattern of the participants in a stepwise fashion and to achieve sustainable lifestyle change. If required, after the basic programme each participant can be included in one of additional programmes, namely the relapse prevention programme (group and individual sessions) or the additional programme (only individual sessions). A total of 13 lifestyle coaches, who had completed a postgraduate training course at the Dutch Academy for Lifestyle and Health (AVLEG), were involved in the pilot programme. More information about the content of the programme can be found elsewhere, as well as the methods, techniques and working approaches used in the intervention [19, 23].

The pilot started in two regions in the southern part of the Netherlands with the programme for adults (Regions 1 and 2) and in two other regions for the children’s programme Regions 3 and 4) in 2014. During the subsequent pilot period, more regions were added. At the end of the study period, the adult programme was implemented in five regions (Regions 1 and 2 plus Regions 5, 6 and 7) and four children’s regions (Regions 3 and 4 plus Regions 2 and 5). The adult participants were mostly referred to CooL by their general practitioners or their practice nurses. The children were mostly referred by the Youth Health Care (YHC) service.

In each region, a project group was responsible for the local implementation of the intervention. These project groups consisted of the central project leader, the lifestyle coaches involved, a coordinator from the local ‘health care group’ (i.e. coordinating organisation for primary care providers) or from the public health services, a representative of the local sports organisation and a care purchasing agent of the health insurance company. During the final year of the study, the role of project leader, which until then had been the responsibility of an external change agent (who was still available in the background), shifted to the main researcher (CvR). In addition to the project groups, there was a steering group which was responsible for general decisions about the programme, its implementation and the evaluation study. During peer feedback meetings with their supervisor, the lifestyle coaches discussed problems that occurred in the implementation, shared best practices and learned from each other’s experiences.

### CooL study

The study protocol of the CooL study has been published and presents a detailed description of the study and the methods [23]. The lifestyle changes achieved among CooL participants have been reported in an earlier publication [19]. Briefly, the results showed positive and sustained changes among adults regarding psychological needs, motivation for physical activity and healthy diet, behaviour-specific barriers, physical activity, dietary behaviours, quality of life and weight. The adult participants lost an average of 2.3 kg after completing the CooL intervention. Among children and their parents, few improvements were found regarding behaviours and quality of life. The children’s BMI z-score (standardized BMI score) did not differ significantly after the intervention.

The present paper describes the implementation process. Both quantitative and qualitative data were used (see Table 1 for an overview). The overall study was designed as an action-oriented study, implying that results of observations are also used as input to improve the content or implementation process of the intervention [24]. Since the main researcher participated in all organisational meetings, this enabled her to observe and simultaneously support the implementation process. The CFIR framework was used to analyse and cluster the data. Data collection took place between 1 May 2014 and 1 April 2017.

**Table 1:** Study components and methods used, for each domain of the Consolidated Framework for Implementation Research

Domain	Evaluation components	Method	Target group	N
Intervention	Evolution of the programme	Observations	Peer feedback meetings	13
	Programme fidelity: executed as intended	Interviews	Lifestyle coaches	12
Lifestyle coaches	Competences of lifestyle coaches	Questionnaire	Lifestyle coaches	13
		Questionnaire	Participants	187
	Tasks of lifestyle coaches	Questionnaire	Referrers, project group members, lifestyle coaches and local parties	129
Inner setting	Organisations in the various CooL regions	Weekly telephone meetings	Project leader	72
Outer setting	Number of referrals and attendance rates	Registration lists and attendance lists	Lifestyle coaches	13
	Involvement and opinion of stakeholders	Questionnaire	Referrers, project group members, lifestyle coaches and local parties	129
Process	Experiences with the programme: satisfaction	Questionnaire	Participants	187
		Group interviews	Participants	6
		Interviews	Participants	4
			Referrers	52
			Project group members	14
			Lifestyle coaches	12
	Facilitating and impeding factors for successful implementation during different implementation phases	Interviews	Referrers	52
			Project group members	14
			Lifestyle coaches	12
		Observations and minutes	Project & steering group & peer feedback meetings	107
			Group sessions	28
		Questionnaire	Referrers, project group members, lifestyle coaches and local parties	129

N = number of respondents

The quantitative measures included the number of referrals, attendance lists of participants, questionnaires for participants about their satisfaction with the intervention and the guidance provided by their lifestyle coach, and questionnaires for lifestyle coaches to assess their competences.

The qualitative methods consisted of group and individual interviews with the participants, lifestyle coaches and other stakeholders, observations and minutes of group sessions and meetings, and a questionnaire with additional process questions. The interview structures were based on various implementation theories [5, 25], adapted to the CooL intervention (see Additional file 2). The interview structures were translated into our coding scheme, while adding additional topics which were concluded out of the interviews. The topics of the interviews were their functioning (interviews with lifestyle coaches and participants), the process of the referral process (interviews with referrers), the implementation process (interviews with project group members and project steering group members) and their opinions about the intervention (interviews with all target groups).

## Results

The results are described for each of the domains presented in Fig. 1, based on the CFIR framework. The facilitating and impeding factors are outlined in each domain and are listed in Table 2. For each domain, multiple perspectives are presented, such as those of the lifestyle coaches, project group members, referrers and participants.

**Table 2:** Facilitating and impeding factors for each domain

Domain	Facilitating factors	Impeding factors
Lifestyle coaches	- High level of work engagement - Empathising with others - Good contacts and getting along with the participants - High involvement - Great enthusiasm - Openness - Supporting instead of directing participants - Patience - Confidence in participants - Knowledge and skills regarding systematic behaviour change	- Lack of entrepreneurship - Lack of networking skills - Not using the professional network for referring
Inner setting	- Having project groups - Locations in the neighbourhood - Support from the health care centre - Cooperation between the LSCs	- No appropriate financial compensation for lifestyle coaches - Too many unpaid administrative tasks for lifestyle coaches
Outer setting		
*Participants*	- Low drop-out rates - Intrinsic motivation to change before the start - High self-efficacy to change	- History of multiple failures in trying to lose weight - Having other more important problems decreases motivation - Financial problems - Sense of not fitting in with the group - Unsupportive parents regarding changing their child’s lifestyle
*Referrers*	- Personal motivation of referrers - Referrers’ knowledge of and experience with lifestyle coaching and the coaches	- Perceived lack of time or priority to be involved in the programme - Some referrers knew too little about the programme
*Context*	- Expected future coverage of CLIs by health insurance - Collaborating with other partners and different disciplines - Increased familiarity with the lifestyle coaches and their role	- Health care professional’s unawareness about their role in lifestyle change
Implementation process		
*Planning*	- Involvement of stakeholders in project groups	- Too little time for implementation to create support among the referrers
*Engaging*	- Creating support - Kick-off meetings - Protocols for lifestyle coaches and referrers	- Not having the logistics organised at the start of the implementation
*Executing*	- Effective communication and collaboration between lifestyle coaches and referrers - Attending more meetings to inform the referrers - Articles in local newspapers	- Time investment for lifestyle coaches, stakeholders and participants - Too few personal contacts with referrers - Lack of clear communication materials
*Reflecting and evaluating*	- Most participants were satisfied - Ensuring well-organised preconditions - Having suitable manuals for new lifestyle coaches	- Too heterogeneous groups and large differences between participants - Too much time between contact moments, and between registration and start of the group - Too few individual coaching sessions (for children) - No ambassador in every region
CooL intervention	- Frequent contacts over a period of six months - Optimised combination of individual and group sessions - Not only focusing on nutrition, but multiple themes including stress and sleep - Learning from peers - Whole family takes part in the children’s programme - Home visits for children - Participant-centred approach - Positive approach aimed at increasing autonomous motivation - Knowledge transfer and practical implications for daily life - Approach tailored to the participants’ needs - Flexibility in design and content - Easily accessible for participants - No charge for participants	- Inadequate time slots for group sessions - Strict inclusion criteria - Participant materials with too much text - Materials not suitable for non-Dutch speaking persons

### Unadapted intervention

At the start of the implementation process, the content of the various programmes was not designed or protocolled in full detail. This left the lifestyle coaches the opportunity to fill in the contents according to their own preferred working methods. Major topics were established in advance as key elements of the programme (physical activity, dietary behaviours, sleep, stress management and behavioural change). The lifestyle coaches were trained to develop their own programme, based on evidence-based behaviour change approaches, their general coaching styles, specific coaching strategies and knowledge gained in their training course.

### Lifestyle coaches

The questionnaire regarding the lifestyle coaches’ competences showed that the coaches were significantly more engaged in their work than average Dutch employees [26]. Empathising with others was their strongest competence, which they also indicated as the most important competence for a lifestyle coach. The coaches evaluated entrepreneurship as their weakest competence, but at the same time they thought this was the least important competence to have as a lifestyle coach. The majority of lifestyle coaches appeared to lack these additional skills during the pilot, which impeded the effectiveness of their coaching. During the interviews, the coaches indicated that coaching skills (i.e. skills to enhance participants autonomous motivation and capability to take-up and self-manage a healthy lifestyle) and empathic skills are necessary.

#### Stakeholders’ perspective

The stakeholders of the intervention network, including referrers, project group members, health insurer, lifestyle coaches and local parties (e.g. local sports clubs and neighbourhood sports coaches) most commonly defined the lifestyle coaches’ tasks as guiding participants towards a sustained healthier lifestyle, addressing all lifestyle themes (such as physical activity and stress management). When asked for more details, they explained they were referring to creating awareness, transferring knowledge, providing information and advice, intrinsically motivating participants, signalling problems, helping participants set realistic goals, supporting, helping participants to learn new skills, and improving self-management. They also emphasised the importance of having a positive approach, monitoring the process, tailoring the programme and finding a suitable form of physical activity together with each participant. Some of the stakeholders mentioned that lifestyle coaches’ tasks also included communicating with referrers, providing them with feedback, referring participants to other professionals and networking with stakeholders.

### Inner setting

#### Financial organisation

The lifestyle coaches, as well as the project group members, had to invest time and money at the start. The health insurance company paid the expenses of the lifestyle coaches in this pilot study. The fees for each individual participant did, however, not cover all the costs for the lifestyle coaches. The meeting time and contact time with absent participants were not included in these fees, nor was the time needed to design the detailed content of the programme and complete portfolios and plans of action. At least eight participants per group were required to break even and make it viable to start a group. For the children’s groups, it was not easy to make up a group large enough to cover the costs.

#### Organisation within regions

In most of the regions, one or two lifestyle coaches were assigned, in which case they both counselled their own groups. In Region 6 the two lifestyle coaches divided the tasks: one coach was responsible for the coaching and the other for the networking and registration of participants. They both experienced this as a good and pleasant task division. In Region 5 the lifestyle coach received administrative support from the local health care group, which helped considerably.

#### Locations

It was a barrier for participants when the meeting location was not in their immediate neighbourhood. Therefore, the group sessions were held in locations as close to the participants residences as possible, and in rent-free or cheap locations, to minimise the intervention costs. The chosen locations included meeting rooms of the health care groups or the health insurance company, community centres and schools. The children’s lifestyle coach of Region 2 was sometimes present at the location of the YHC referrer. This gave the participants the opportunity to immediately plan an intake session (i.e. the first session of the intervention to check the participants’ motivation and to investigate their treatment demand).

### Outer setting

#### Participants

During the study period, 494 adults were referred to the CooL intervention, 358 of whom actually started the intervention. A total of 66 adults (18%) dropped out during the programme. The number of referrals of children and adolescents was 192, 106 of whom started the programme, and 22 (21%) children dropped out.

##### Participants’ characteristics

Among the CooL participants, adults had an average BMI of 36.1, while the children had an average BMI z-score of 2.3. The self-reported educational level of the majority of the adults and the children’s parents was low or intermediate. The study population had tried to lose weight before, but were unable to maintain this weight loss for more than 1 year. Participants with a low autonomous motivation were more likely to drop out of the programme. The lifestyle coaches noticed during the implementation that the participant’s motivation should preferably be checked at the intake session, which made the operationalisation of the inclusion criteria stricter as the pilot progressed. Participants with a higher autonomous motivation were more conscious of their unhealthy behaviours and felt more responsible for them. Overweight parents were less motivated to participate in the programme with their children, compared to parents with a normal weight. In the baseline questionnaire, 15% of the parents answered that it had actually come as a surprise to them that their child’s weight was a matter of concern.

##### Reasons and criteria for not starting

There were several reasons why potential participants decided not to attend the programme. The most frequently mentioned reason was lack of motivation (e.g. lack of interest to start with CooL). This appeared to be more often the case for participants with multiple problems, such as diseases, financial problems or mental problems. Another important impeding factor was that some participants did not like to participate in a group. Most children or their parents showed a need for more individual guidance, which was sometimes provided by the lifestyle coaches.

The most common criticism among lifestyle coaches and referrers was the strictness of the inclusion criteria for CooL, particularly for children. When children were obese at a young age, this usually meant there were more problems in the family. We found that in these multi-problem families, lifestyle change is typically not their first priority. Lifestyle coaches reported a preference for a less strict inclusion criterion for weight status.

#### Referrers

Referrers reported that patients were hard to reach and to motivate for participation in the programme. Furthermore, they saw multiple barriers to taking part in the intervention, especially at the beginning. They were under great pressure of time, and they felt that there was no time or priority for referring patients to the programme. Their awareness of the intervention decreased over time, because they were not referring to it on a regular basis. Professionals who saw the advantage of the intervention and had a passion for prevention referred more patients. It depended on the region and the lifestyle coach’s place within the care network whether they received more referrals and support from the referrers. Considerable time went by before referrers became aware of the positive results of the intervention and realised the benefits and relevance of the CooL programme.

#### Context

The goal of the pilot was to evaluate and further develop the implementation process. The goal for Centraal Ziekenfonds (CZ) health insurance company, was to develop an optimal system for the reimbursement of CLIs by health insurance companies, with the ultimate aim of reducing the health care costs in the longer term. At the time the pilot started, in 2014, obesity care was not a common theme to discuss during consultations in primary care [27]. General practitioners were insufficiently trained to discuss lifestyle with their patients [28]. Health care professionals typically applied a mono-disciplinary approach to their patients, for example physiotherapists mainly tried to improve their musculoskeletal system [29]. Care for patients was fragmented. The idea that obesity should be addressed in an integrated approach did gain some ground, but at a very slow pace [20]. At the local level, the implementation of CooL started in regions where covenants, connections and other arrangements among the care providers already existed and prevention was already on the agenda more explicitly than in many other regions in the country.

##### The central role of lifestyle coaches

In the course of the process, the lifestyle coaches’ role as linchpins in obesity care appeared crucial. If lifestyle coaches were part of relatively dense networks (i.e. when they had more ties and connections with stakeholders), this meant that participants were more likely to be referred to these coaches. In any case, referral to CooL was suboptimal and lifestyle coaches should become more visible as an important stakeholder in obesity care.

##### Changed context

Currently, health care professionals and policy makers have become more aware of the importance of lifestyle behaviour for health outcomes [28]. Integrated approaches to the prevention of chronic diseases have become more common over time. In the course of the implementation process it was becoming clearer that CLIs would be included in health insurance policies in the Netherlands from 2019 onwards [30]. This had a positive influence on the motivation of the lifestyle coaches, referrers and other stakeholders. The lifestyle coaches invested more time in describing and detailing the adjusted intervention contents than in the early stages of the pilot. The referrers increasingly perceived CooL as a permanent referral option instead of just another project.

##### Stakeholders’ contributions

Most stakeholders (66%) reported themselves as contributing relatively little to the programme; although some stakeholders were relatively active (24%) and a small proportion contributed greatly (10%). The most commonly mentioned reasons to participate were: improving the participants’ health (82%); the sense that the programme was a good initiative (70%); collaboration with other disciplines/organisations (33%); and referring people (28%). Furthermore, 46% fully agreed (on a 5-point Likert scale) with the statement that the lifestyle coach represented a useful addition to the health care network and 48% fully agreed that the lifestyle coaching programme was a valuable innovation.

### Implementation process

#### Planning

The implementation started with the programme for adults, and involved a small selection of interested general practices. Meanwhile, the sample size was calculated and lifestyle coaches were spread over the regions. When the number of referrals was found to be low, the inclusion period was extended and all general practices in each region were invited to refer patients to CooL. Some practices (2%) declined this invitation, as they did not want to invest time.

#### Engaging

The lifestyle coaches used kick-off meetings and information provision to referrers during group or individual meetings to try and create more support among the referrers. The referrers received an information package with a flyer for patients and a referral protocol, which presented information on how to sign up patients and what was expected from them. The lifestyle coaches had also been informed about the referral process and the execution of the intervention by means of a protocol.

In the beginning of the pilot programme, the logistics of the intervention had not yet been fully organised at the start of the intervention’s implementation. The contacts with stakeholders had already been established before the information was prepared and the programme was finalised. On the one hand, this meant that the information was distributed in phases. On the other hand, the stakeholders could already contribute to the implementation process.

When one of the two programmes had already been implemented in a particular region, the chances were greater that the second programme would be implemented as well (in most cases the children’s programme followed the adult programme).

#### Executing

The referrers indicated during the interviews that they wanted to know who the lifestyle coaches were, and the lifestyle coaches noticed that the referrers had many practical questions. Project groups members therefore pointed out that personal contact was very important to increase the referrers’ motivation. This demanded a lot of time investment on the part of the lifestyle coaches. Furthermore, the question remained to what extent the referrers were aware of the programme and the referral process. In each region, newsletters were sent by the health care group or public health services, presenting the most important information and updates.

### Reflecting and evaluating

Based on attendance lists, it appeared that the adult participants attended on average 5.3 (±2.3) group sessions and 2.9 (±0.9) hours of individual coaching. Their total programme covered 188.4 (±89.4) days. Children, adolescents and their parents participated in the CooL programme for 229.4 (±128.5) days. They attended 3.8 (±2.6) group sessions and had 4.2 (±1.9) hours of individual sessions.

#### Evaluation by the participants

On average, the participants were satisfied with the programme, the group sessions, individual sessions and the work of the lifestyle coach. The participants rated the programme at about 8 out of 10 (adults: 8.6; parents: 8.5; children: 7.8). There were a few exceptions. For example, some participants had expected a stricter approach, in which they were told how much to exercise and what to eat. This expectation conflicted directly with the nature of lifestyle coaching, in which the participant is supposed to take the leading role and is in charge of their own goals and corresponding actions.

Most participants perceived the combination of group and individual sessions as pleasant. The individual guidance enabled them to discuss personal problems. The group dynamics in the group sessions linked them to fellow sufferers and familiar problems were discussed. However, some participants reported in the questionnaire that they felt a need for a more personal approach. This remark typically came from participants in larger groups (often larger than ten members) and from participants in groups with persons with special needs (e.g. persons with a mental disorder). This made it harder to give enough personal time and space to all the group members.

In addition, some of the participants wanted to have less written and more practical assignments, for example more assignments with pictures, audio-visual tools and digital materials. These alternatives could replace the text that was used in the materials. Finally, the participants mentioned in the early stage of the pilot programme that they needed refresher sessions to better maintain their changed behaviours.

#### Lifestyle coaches’ perspective

According to the lifestyle coaches, the ideal group size was about ten to twelve participants. In reality, the groups were often smaller, since some of the participants did not always attend. Moreover, it was hard to get enough people for the groups, which made the time between registration and the start of the programme rather long for some participants. It also led to mixed group compositions, with different ages and cognitive skills. Participants could not identify themselves with the other group members when the differences between them were large. The lifestyle coaches argued that it would be desirable to work with more homogeneous groups, so they could easily adjust the content of the programme to the level of the group. The participants could then learn more from each other and the group process would improve. Furthermore, the lifestyle coaches perceived the home visits for children and their parents as valuable, as it made their daily lives and behavioural patterns more visible and could be discussed more easily.

#### Project groups

The implementation process was discussed at every monthly project group meeting in each region. If the implementation was not yet successful, new actions were instigated to improve the information available among the stakeholders. In the early stage of the pilot, the project group members noticed that the division of roles and expectations was not clear to all of them. In some cases, it was unclear who was responsible for which tasks, such as arranging the location for the group sessions. Another observation was that the project leaders were often geographically far removed from the pilot region and that they were not familiar with the stakeholders in the networks.

### Adapted intervention

The core components of the programme were the sessions with their fixed themes (see Additional file 1: Table S3), as well as appointing one lifestyle coach to each group. The themes were sometimes presented in a different order and the contents were adapted to meet the needs of the group. The exact content and the practical exercises were part of the ‘adaptable periphery’ of the intervention protocol. In the unadapted intervention, the lifestyle coaches started with their own custom-made content and exercises. During the course of the implementation process, the lifestyle coaches shaped and finalised the content based on their professional knowledge, their experience, feedback from the participants, evaluation sessions with other lifestyle coaches and interim findings from the current action-oriented study. They exchanged practical exercises and assignments for the group sessions during peer feedback meetings. Gradually during the study period, they combined their best practices into a final programme format. A document was produced which described the goals and multiple examples of exercises for each group session, to support lifestyle coaches in designing sessions for their own groups and in their own context. When the intervention document was being drafted, the coaches were invited to substantiate the programme with underlying theories, strategies and applications [31].

## Discussion

The aim of this study was to examine the implementation process of the CooL intervention and its facilitating and impeding factors. We found that the principles that contributed most to the successful implementation of CooL were: having one professional (the lifestyle coach) for multiple lifestyle-related themes, offering a combination of group and individual sessions for adults, the family approach for children, a high frequency of sessions, easy accessibility for participants and the fact that the programme was offered free of charge. Impeding aspects for the intervention were the strict inclusion criteria and small group sizes. Crucial factors for lifestyle coaches included empathising with the participants and having a high work engagement. Impeding factors for the lifestyle coaches were a lack of networking skills and entrepreneurship. The most important facilitating factors for the inner setting (i.e. the CooL organisation) were the project groups and close proximity of the intervention location. CooL participants were more likely to participate when they had a strong intrinsic motivation to change. Factors that make it less likely for people to participate or to complete the programme included not fitting in with the group and having financial constraints. As regards the outer setting, the contacts between lifestyle coaches and their network were crucial. Greater familiarity with and a positive attitude towards the lifestyle coaches’ role among the stakeholders were necessary for effective implementation. It helped if the coaches were able to strengthen their network to ensure optimal referral of participants.

Effective implementation starts by creating support among stakeholders, such as referrers. Since the role of lifestyle coach is a new one in the health care system, it has not yet become very familiar. Therefore, we recommend that the central role of the lifestyle coach is more clearly positioned in the integrated approach to obesity. Above all, personal contacts are crucial, and intensive collaboration between coaches and other professionals will help increase their familiarity and trust among other network members [32]. A trend towards increased motivation of referrers was observed towards the end of the pilot period.

If more stakeholders support the intervention, they will probably contribute more effectively to accelerating the recruitment of participants. Slow recruitment processes have also been found in other studies [7, 33] and this remains an issue of concern. An important cause of the low number of referrals was the lack of clear communication materials for the referrers. In combination with the low frequency of personal contacts with referrers, this meant that not all referrers had sufficient knowledge about the intervention, about their specific role in the process and about how to refer patients. More contacts and better information could probably take away the barriers from the referrers, such as the time investment required for referring [34]. Since general practitioners are not trained to assess a patient’s motivation, they should be assisted by the lifestyle coaches to make this assessment [35]. The fact that the costs of CLIs are expected to be covered by health insurance may help to institutionalise the referral process [30]. If lifestyle coaches informed the referrers more effectively about the participants’ progress, referrers might take a more positive view of the programme [36, 37].

Investment in the contacts among the stakeholders could make the relationships sustainable, with help from an ‘ambassador’ or a broker [16, 38]. Such an ambassador should be in close contact with the stakeholders in the region and can probably take over some of the networking and entrepreneurial tasks from the lifestyle coach, if this person is not the lifestyle coach. The role could be filled by the lifestyle coach, a local project leader, someone from a central organisation (e.g. a health care group) or a central person in the network of public health and health care (e.g. a health broker [39]).

Extensive preparation and implementation time are needed to create support among the stakeholders to engage them with the program and to create an optimal intervention context. This is often underestimated. Depending on the characteristics of the context, it can take up to a few years [40].

The autonomous motivation to change has been shown to be crucial for the attendance of participants as well as for intervention effects [16, 41]. People differ in their readiness to change their unhealthy behaviour. This depends on their previous experiences [42] and the extent to which they experience the negative effects of their current behaviour. In order to increase this autonomous motivation the lifestyle coach uses methods such as motivational interviewing [43].

### Strengths and limitations of the study

Strengths of this study were its action-oriented approach, the real-world setting in different regions and the use of several implementation process methods and instruments. Thanks to the action-oriented approach, the collaboration between the lifestyle coaches and the researchers was good and the implementation process could be closely followed and improved when needed. Implementing an intervention in a real-world setting is always complex, due to contextual and systemic processes [44]. But the chances of achieving sustainability of the CooL intervention and its nation-wide dissemination are probably greater than if the pilot had been accompanied by a controlled trial [45]. Another added value of this study was the use of mixed methods, which gave us information from different points of view, viz. those of the stakeholders, lifestyle coaches, researchers and participants.

The lifestyle coaches constantly adjusted and adapted the CooL programme to the participants’ needs during the study period. They worked in their own way, but used the same themes, general principles and way of thinking. These programme changes and the different ways in which it was executed made it impossible to measure the programme fidelity among the lifestyle coaches. This may be viewed as a limitation, but in line with basic assumptions underlying the CFIR for evaluating interventions in complex systems [46, 47], we postulate that adaptation is desirable and promoting complete programme fidelity may even be harmful (Schaap et al., unpublished observations). A limitation of this study is that the data were not analysed with qualitative software programmes, such as Nvivo. The amount of data and the different types of qualitative data (ranging from observations and minutes of meetings to semi-structured interviews) prevented us from adopting a computerised approach to the analyses.

## Conclusions

The aim of this study was to examine the implementation process of the CooL intervention and its facilitating and impeding factors. A substantial number of barriers have been overcome and promising opportunities have arisen for integrating lifestyle coaching in a broader approach, to bridge the gap between prevention and treatment of chronic diseases. However, the dissemination process of CooL still needs to be improved further. Networking activities should be intensified and the contents of the intervention continuously improved to fit both the inner and outer implementation settings. It will take time before the lifestyle coaches have become accepted as valuable professionals who bridge the gap between the public health sector and health care settings. We expect our recommendations to be helpful in improving the dissemination and monitoring of combined lifestyle interventions.

## Additional files

Additional file 1: **Table S3.** Number of sessions per target group and per programme, and themes per group session. (DOCX 15 kb)

Additional file 2: Interview guides. (DOCX 28 kb)

## Data Availability

The questionnaires and interview data are available in Dutch from the corresponding author upon reasonable request.

## Abbreviations

CFIR: Consolidated Framework for Implementation Research
CLI: Combined lifestyle interventions
CooL: Coaching on Lifestyle
YHC: Youth Health Care
