# Supplementary material for: The implementation of the coaching on lifestyle (CooL) intervention: lessons learnt
Source: BMC Health Serv Res. 2019 Sep 14;19:667. doi: 10.1186/s12913-019-4457-7 (PMC6744697; doi:10.1186/s12913-019-4457-7)
Supplement: Supplementary file 1 — Table S3. Number of sessions per target group and per programme, and themes per group session. (DOCX 15 kb) [file 12913_2019_4457_MOESM1_ESM.docx]

# Appendix 1. Additional table

**Table 3** Number of sessions per target group and per programme, and themes per group session

| **Components** | **Children** | **Adolescents** | **Adults** |
| --- | --- | --- | --- |
| Basic programme | 9 to 10 months | 9 to 10 months | 7.5 months |
| *Individual sessions* | Maximum 7 hours (10x) at home | Maximum 7 hours (10x) at home | 2x 60 minutes &  2x 45 minutes |
| *Group sessions* | 8x 90 minutes for the parents | 5x 90 minutes for adolescents  1x 90 minutes for parents  2x 90 minutes for adolescents and parents | 8x 90 minutes |
| *1* | Awareness and behaviour change | Awareness and behaviour change | Awareness and behaviour change |
| *2* | Physical activity | Acting as a role model (only for parents) | Physical activity |
| *3* | Nutrition | Physical activity | Structured eating patterns |
| *4* | Setting boundaries and rewarding | Nutrition (including parents) | Sleep, relaxing, stresses |
| *5* | Acting as a role model | Snacking | Time management |
| *6* | Sleep, relaxing, stresses | Sleep and relaxing | Pitfalls |
| *7* | Pitfalls and planning | Stresses and pitfalls | Relapse prevention |
| *8* | Self-regulation for the family | Self-regulation for the family (including parents) | Self-regulation |
| Relapse prevention programme | Same number of sessions as basic intervention, spread over 2 years | Same number of sessions as basic intervention, spread over 2 years | Same number of sessions as basic intervention, spread over 2 years |
| Additional programme | - | - | 10x 30 minutes individual sessions |
