# Supplementary material for: The implementation of the coaching on lifestyle (CooL) intervention: lessons learnt
Source: BMC Health Serv Res. 2019 Sep 14;19:667. doi: 10.1186/s12913-019-4457-7 (PMC6744697; doi:10.1186/s12913-019-4457-7)
Supplement: Supplementary file 2 — Interview guides. (DOCX 28 kb) [file 12913_2019_4457_MOESM2_ESM.docx]

# **Appendix 2. Interview guides**

**Lifestyle coaches**

- Your contribution to the CooL pilot
  - What are the most important reasons for you to participate in the CooL pilot?
  - What is it like to participate in this pilot as a lifestyle coach?
- Facilitating and impeding factors
  - What do you like/dislike about your job as a lifestyle coach?
  - What factors in general influence your work? Can you give positive and negative examples? Which impeding factors do you come up against when performing your work?
  - What needs to be changed to reduce the negative factors?
- Competences
  - Which competences or skills do you need as a lifestyle coach?
  - Do you consider yourself a good lifestyle coach?
  - If so, what makes you a good lifestyle coach, and what would you need to improve?
  - How would you describe your coaching style?
- Lifestyle coaches’ role
  - What are the activities you perform as part of your job?
  - What tasks do you think a lifestyle coach should perform?
  - Should a lifestyle coach strive to obtain a more visible position within the health care network?
  - What should the position of the lifestyle coach within the health care network look like?
  - How do you see the future of lifestyle coaches?
  - How can the CooL intervention be implemented in the rest of the Netherlands and by new lifestyle coaches?
- Target group of CooL
  - For what reasons did people want to participate in the intervention?
  - To what extent did participants have multiple problems?
  - Was the Cool intervention the right intervention to solve these problems?
  - In how many cases did you refer participants to other professionals and why?
- Maintenance of participants’ new lifestyle
  - Can you identify a certain type of participant of whom you would expect that they could maintain their lifestyle change?
  - What are reasons for a relapse?
  - Can this be prevented and how?
  - What should be changed in the intervention to prevent relapses?
- Referral process
  - What is your experience of the communication with the referrers and of the referral process?
  - Did you refer any participants to local sports organisations?
  - What were facilitating and impeding factors regarding the recruitment and referral process of potential participants?
- CooL intervention development
  - How was the programme developed?
  - What are the essential elements of the intervention?
  - How did you develop and prepare your own sessions and exercises?
  - What was your experience of the preparation of the programme?
- Recommendations for the intervention
  - How satisfied are you with the intervention?
  - Do you have recommendations for improving the intervention?
- Programme execution
  - To what extent did you manage to address the essential elements in the sessions?
  - To what extent were you able to carry out the complete programme that was planned for each session?
  - Which programme components did you adjust (per session), and why?
  - Does the way the programme was carried out match your preferred way of working?
  - Did problems with regard to the programme implementation arise over time?

**Project group members**

- Own contribution
  - What are the most important reasons for you to participate in the CooL pilot study?
  - How intensively do you contribute to the execution of the CooL intervention?
  - How much investment does it take you/your organisation to participate in the pilot?
- Starting phase
  - How was the pilot started? Who came up with the initiative to start the programme?
  - When were you/was your organisation approached to participate in the pilot?
  - Why was the CooL intervention chosen?
- Intervention planning phase
  - Can you elaborate about what happened after the starting phase to enable the CooL intervention to be carried out?
  - How were the regions and lifestyle coaches selected?
  - How were the required competences of the lifestyle coaches determined?
  - How was the remuneration of the lifestyle coaches calculated?
- Intervention development
  - How was the exercise part of the intervention developed?
  - How was the intervention protocol, including the content of the group sessions, created?
  - What are the essential elements of the intervention?
  - How was the referral process set up?
- Lifestyle coaches’ role
  - What do you think about the functioning of the lifestyle coaches?
  - What tasks do you think a lifestyle coach should perform?
  - Do you think that lifestyle coaches offer any added value to the health care system?
  - How do you see the role of lifestyle coaches ideally within the health care network?
- Implementation
  - Was it clear from the start how the intervention would be implemented?
  - Who determined the changes (if any) in the intervention or the referral process? And who decided to implement the changes in that way?
  - Were the changes an improvement on how it was initially?
  - Did you agree with the way the intervention and the referral process were planned?
  - How were referrers invited to participate in the pilot?
- Execution
  - What do you think are the primary reasons for the low number of referrals?
  - What is your experience of the project/steering group meetings?
- Evaluation
  - What went well and what did not go well during the entire process?
  - What were the impeding factors and what could be improved? What could have been done differently?
  - How should the implementation of such an intervention proceed ideally?
- Future
  - To what extent do you think the combined lifestyle interventions will be reimbursed by the basic health insurance?
  - How do you think the intervention will be continued after the pilot?

**Participants**

- Referral
  - Who introduced the programme to you?
  - What was the main reason for you to participate?
  - Was there something that made you doubt about starting the programme (e.g. group participation or timing)?
  - How did the referral to the lifestyle coach go? How did you experience this?
  - How long did it take before you had contact with the lifestyle coach before the intake took place?
  - What do you think are points for improvement regarding the referral?
- Feasibility
  - Was it feasible for you to complete the programme? Were you able to attend all the sessions? If not, why not, e.g. because of the distance or time?
  - How high was your motivation to participate in the programme?
  - To what extent have you participated in this programme and prepared yourself?
  - Was there a moment when you wanted to stop taking part in the programme?
  - Would you still like to participate in the programme if you had to pay for it?
- Your lifestyle coach
  - What did you think about your lifestyle coach? What was positive, what could be improved?
  - How would you describe the coaching by your lifestyle coach?
  - Did the way she worked suit you well?
  - Which competences/skills should a good lifestyle coach have?
  - Do you have any tips for your lifestyle coach to improve?
- Experiences with the intervention
  - What was your experience of the programme? What is your general impression of the programme?
  - How would you explain the programme to acquaintances?
  - What appealed to you most about the programme?
  - What are the most important elements of the programme?
  - Which part did you benefit the most from? Why?
  - Which part did you benefit least from? Why?
  - What did you think about the different themes during the group meetings?
  - Did the themes match your needs/problems?
  - What did you think of the workbook and the homework assignments? What could have been different?
  - How did you like being in a group?
  - What did you think of the individual and group sessions?
  - What did you think of the session with someone from the local sports organisation?
  - To what extent did the programme meet your expectations?
  - Do you have any ideas for improving the programme?
  - To what extent would you like the programme to be tailored to your own preferences and circumstances?
  - Do you think the right target group was included?
- Outcome
  - Have you developed a healthier lifestyle as a result of to the programme?
  - What did you learn about adjusting your diet and physical activity level during the programme?
  - What did you hope to achieve with this programme? Have you reached this goal?
  - To what extent are you satisfied with what you have achieved?
  - How would you describe your progress in the programme to your practice nurse?
  - What has helped you most to achieve these results?
  - What did your social environment think about you participating in the programme and about changing your lifestyle?
  - To what extent do you think you can maintain the new lifestyle without the lifestyle coach?
- Follow-up process
  - Are you going to attend one of the follow-up programmes?
  - Why did you, or did you not, opt ​​for this?

**Referrers**

- Own contribution
  - What are the most important reasons for you to participate in the CooL pilot?
  - What is your motivation to participate in this pilot?
  - How intensively do you contribute to the CooL intervention?
  - How much investment does it take you/your organisation to participate in the pilot?
  - To what extent do your colleagues show interest and commitment in the CooL intervention and referral of participants to it?
- Lifestyle coaches’ role
  - What do you think about the functioning of the lifestyle coaches?
  - What tasks do you think a lifestyle coach should perform?
  - Do you think that lifestyle coaches offer any added value to the health care system?
  - How do you see the role of a lifestyle coach ideally within the health care network?
- Referring process
  - Have you referred any patients to CooL?
    - If yes:
      - Is it clear to you who you can refer to CooL?
      - What was your experience of this?
      - When do you refer a patient?
      - How do you go about starting to talk about this topic?
      - Do you feel confident about explaining the intervention?
    - If not: have you tried this?
  - What, in your opinion, are the facilitating and impeding factors for a successful referral to the lifestyle coach intervention?
- Communication
  - What was your experience of the communication about the CooL intervention?
  - To what extent did you (at the start) receive sufficient information about the CooL intervention?
- Recommendations for the intervention
  - How satisfied are you with the intervention?
  - Do you have recommendations for improving the intervention?
